# What Motivates People to Receive Continuous COVID-19 Vaccine Booster Shots? An Expectation Confirmation Theory Perspective

**DOI:** 10.3390/healthcare10122535

**Published:** 2022-12-14

**Authors:** Jingfang Liu, Shuangjinhua Lu, Caiying Lu

**Affiliations:** School of Management, Shanghai University, No. 20, Chengzhong Road, Jiading District, Shanghai 201899, China

**Keywords:** COVID-19, SARS-CoV-2, booster dose, booster shot, expectation confirmation theory, vaccination intention, China, structural equation model

## Abstract

(1) Background: Abundant evidence has shown that the COVID-19 vaccine booster is highly effective against the Omicron variant. It is of great practical significance to explore the factors influencing the intention to receive COVID-19 booster shots. (2) Methods: We introduced expectation confirmation theory as the basis to construct a model of the factors of the vaccination intention for COVID-19 vaccine boosters. We obtained two batches of questionnaires through Chinese social platforms, with a valid sample size of 572. To test the model, we used SmartPLS3.0 software for empirical analysis. (3) Results: In terms of the characteristics of the vaccine itself, perceived vaccine efficacy and perceived vaccine safety had significant positive effects on expectation confirmation. Regarding vaccination services, perceived vaccination convenience also had a significant positive effect on expectation confirmation. Expectation confirmation positively affected the vaccination intention for the COVID-19 vaccine boosters. Furthermore, the results showed two moderating effects: first, health consciousness negatively moderated the positive effect of perceived vaccine safety on expectation confirmation; second, the time interval since the last dose negatively moderated the positive effect of perceived vaccine efficacy on expectation confirmation. (4) Conclusions: Our research demonstrated that there is an expectation confirmation process for previous COVID-19 vaccines before people consider whether to obtain a booster shot. Perceived vaccine efficacy and perceived vaccine safety remained important factors in receiving COVID-19 booster shots, and our conclusions were consistent with previous literature. In this study, multiple dimensions such as distance and cost were used to measure perceived vaccination convenience. This new variable improve the explanatory power of the convenience of the vaccination service and enrich the variables of the factor model of vaccination intention. In addition, the moderating effects of health consciousness and time interval were found. The findings can provide a theoretical reference for public health institutions to help them understand the formation process of people’s intention to receive the COVID-19 vaccine booster.

## 1. Introduction

### 1.1. Background

The COVID-19 pandemic continues to unsettle the entire world, and it has had an unprecedented impact on daily life, the economy, and other aspects of human society. According to The World Health Organization (WHO), as of May 2022, over 500 million confirmed cases and over six million deaths have been reported globally [1]. In the past two years, the WHO has approved a variety of safe and effective COVID-19 vaccines in countries around the world, and the mass vaccination programs continue to be rolled out worldwide. However, on 24 November, 2021, the WHO announced a new severe acute respiratory syndrome coronavirus 2 variant called “Omicron”. It is particularly worrying that the Omicron variant has increased infectivity and resistance to vaccine-induced immunity [2]. Breakthrough infections are increasing rapidly globally due to the waning vaccine-induced immunity and the emergence of the Omicron variant [3]. Medical experts recommend that the public maintain high antibody levels by receiving COVID-19 booster shots to help halt the rapid spread of Omicron. A booster shot, also known as a booster immunization, is an additional vaccination given to fully vaccinated people to counter their waning antibodies. As the virus mutates and spreads, a massive COVID-19 vaccine booster program remains our best hope for containing the COVID-19 pandemic [4].

Evidence is mounting that the COVID-19 vaccine booster is highly effective against the Omicron variant. A study reported a significant drop in hospitalizations for COVID-19 among people who received a booster shot. Specifically, there has been an 81% reduction in the risk of hospitalizations after a third shot compared to those who did not receive the booster shot [5]. After receiving the third booster shot, higher levels of antibodies against Omicron were developed, meaning that the protection period lasted longer [6]. Moreover, the efficacy of the third injection was distinctly significant, and the neutralization efficiency after the third injection against the Omicron variant was 100 times higher than the efficacy after the second injection [7].

Many countries and regions have issued relevant policies and plans to call on the public to receive booster shots on time, especially for the elderly, immunocompromised people, and those at higher risk of exposure to COVID-19.A review study systematically reviewed 162 studies on the efficacy of COVID-19 vaccines in immunocompromised populations, most of which confirmed that immunogenicity and efficacy were significantly lower in immunocompromised populations than in healthy populations after full vaccination. Immunocompromised people include patients with different immune diseases such as organ transplantation patients, hematologic malignancies patients, cancer patients, and dialysis patients [8]. In addition, the absence of neutralizing antibodies after vaccination is usually associated with older age, and the same findings were observed in clinical data related to influenza vaccines [9] and hepatitis B vaccines [10]. Several studies have shown that immune-compromised people have increased levels of neutralizing antibodies after a booster shot. Therefore, the targeted strategies presented in these studies emphasize the importance of additional booster injections for special populations [8].

However, vaccine hesitancy and refusal remain significant obstacles to enhancing the vaccination coverage of the COVID-19 vaccine boosters. Therefore, our research question was: what factors affect people’s vaccination intention for COVID-19 vaccine boosters during the pandemic?

### 1.2. The Goals and Innovations of Our Work

To explore this research question, we constructed a research model on the factors of the vaccination intention for COVID-19 vaccine boosters based on expectation confirmation theory. To test this model, we used a questionnaire. Expectation confirmation theory is often used to explain consumers’ “repeated purchase” or “continuous use” of a product or service. Booster shots are additional vaccinations after full vaccinations, which can be analogous to repeated purchases by consumers. Therefore, the vaccination intention for COVID-19 vaccine boosters can also be regarded as revaccination intention. In our model, the vaccination intention for COVID-19 vaccine boosters is a dependent variable, and people form their vaccination intention through the expectation confirmation of perceived vaccine efficacy, perceived vaccination convenience, and perceived vaccine safety. In addition, we found the moderating effects of two variables in the model: health consciousness and the time interval between vaccinations.

The first innovation of this study: We introduced expectation confirmation theory as the basis to construct a model of the factors of vaccination intention for COVID-19 vaccine boosters. During the pandemic, many scholars paid attention to studies on vaccination intention, especially about COVID-19 vaccines. However, previous studies on vaccine intention were based on the theory of planned behavior [11], conspiracy beliefs, and protection motivation theory [12,13]. Few studies on vaccine intention were based on expectation confirmation theory. A previous study based on the health action process approach model, expectation confirmation model, and vaccine hesitancy theory constructed a three-stage model of public motivation, intention, and behavior for COVID-19 vaccination [14], with a wide range of research perspectives. Different from the previous study, our study focuses on the process of the vaccination intention formation of the COVID-19 booster shots.

The second innovation of this study: We introduced perceived vaccination convenience to enhance and improve the explanatory power of the convenience of the vaccination service and enrich the variables of the factor model of vaccination intention.

The third innovation of this study: We found the moderating effect of health consciousness on the relationship between the perceived vaccine safety and the expectation confirmation and the moderating effect of the time interval on the relationship between the perceived vaccine efficacy and the expectation confirmation.

## 2. Literature Review

### 2.1. Studies Related to Vaccination Intention

The research gaps were identified by reviewing previous studies related to vaccination intention. What factors influence people’s opinions and choices about vaccination? It is a distinctly important research question. In the past few decades, scholars worldwide have conducted extensive studies on the factors influencing vaccination intention. These studies attributed the reasons for people’s vaccine hesitancy or refusal to: first, the characteristics of the vaccine itself, such as the information source of the vaccine [15], the vaccine’s safety [16], and the vaccine’s efficacy [16,17]; second, the health status and cognitive level of the vaccine recipients, such as the severity of the disease [18] and the cognition of the disease [19]; third, the factors related to vaccine services, such as vaccine shortages [20] and trust in health professionals [19,21]. In addition, many studies [22,23,24,25,26] confirmed that demographic variables such as sex, age, education, income, and body mass index (BMI) are factors affecting vaccination intention. However, few studies have focused on the convenience of vaccination services. A study found that the geographical distance between home or work and the vaccination site was an essential factor significantly affecting the vaccination intention [27]. Geographical distance is an objective indicator, and we believe that this indicator alone is insufficient to describe the convenience of vaccine services. People’s perception of service convenience is a subjective evaluation formed by multiple dimensions, which should also include less effort and lower cost.

### 2.2. Studies Related to the Vaccination Intention for COVID-19 Vaccine Boosters

Currently, studies on booster shots of the COVID-19 vaccine are mainly focused on immunology and clinical medicine. There are few studies on the intention to receive COVID-19 booster shots and the influencing factors. One study found that education level and vaccine literacy play an essential role in the vaccination intention for COVID-19 vaccine boosters. Disseminating scientific knowledge about the vaccines to people with lower education levels or vaccine knowledge levels is critical to improving booster vaccination coverage [28]. Another study, from the health belief model perspective, revealed that perceived disease susceptibility, perceived benefits, cues to action, perceived barriers, and some demographic variables (such as age, education, and income) were associated with the acceptance of a third COVID-19 vaccine [29]. Much previous evidence has suggested that urban workers are more likely to be vaccinated against COVID-19 than rural workers. A new study on COVID-19 booster shots focused on urban workers. It concluded that urban workers with a strong work organization, high levels of vaccine knowledge, and intensive social capital were more likely to receive COVID-19 booster shots than other workers [30]. A brief report investigated the public’s attitudes among those who had received COVID-19 booster shots. The most common reasons for opposition were doubt about the necessity of booster shots and fear of adverse reactions. In addition, it reported that trust in public health guidance and high education levels significantly influenced the intention to receive a booster vaccination [31]. We found that almost all existing studies on booster vaccination intentions lacked a theoretical framework. Furthermore, the factors found in these studies were not significantly different from those found in general (nonbooster shot) vaccination intentions. In this study, we innovatively introduced the expectation confirmation theory to construct a model of the factors of vaccination intention for COVID-19 vaccine boosters. The model linked people’s previous vaccination experience with the booster vaccination intention to understand people’s decision-making process when faced with booster shots.

### 2.3. Studies Related to Expectation Confirmation Theory

Expectation confirmation theory was proposed by the scholar Oliver in 1980 [32], which originated from the research into customer repurchase intentions. The initial theoretical framework contained five variables: perceived performance, expectation, confirmation, satisfaction, and repurchase intention. Expectation confirmation theory has been widely applied in studies on consumers’ continuous use/purchase behavior, including consumer satisfaction, customer loyalty, and service marketing [33]. In 2001, the scholar Bhattacherjee [34] extended this theory to the related research on the continuous use intention of information systems(IS), involving online scenarios such as social networking sites, information retrieval systems, and mobile commerce. Because expectation was formed by media information and others’ evaluation, Bhattacherjee argued that expectation had a limited impact on users’ continuous intention. Users may have cognitive changes during the process of using, and the expectation confirmation after use integrated the user’s own experience based on the expectation; so, it could replace expectation and become the influencing factor of users’ subsequent decisions. He considered that expectation confirmation was the difference between the user’s experience before and after using a product or system. A high level of expectation confirmation indicated that the user’s expectation after using the product or system was higher than their initial expectation. The expectation confirmation model proposed by Bhattacherjee included four variables: perceived usefulness, expectation confirmation, satisfaction, and continuous intention. This model is more suitable to be applied to a variety of research situations. Therefore, a large number of subsequent studies based on expectation confirmation theory use this model as the basis to construct new models. In the vaccination scenario, expectation confirmation theory has some explanatory power for studies on whether people are willing to obtain booster shots. However, although booster shots have widely existed in immunization procedures for hepatitis [35], influenza [36], and other vaccines, there are few studies on vaccination intention based on expectation confirmation theory.

Different from vaccination against COVID-19 (nonbooster shot), booster vaccination is a “revaccination” behavior. It is of great practical significance to study the factors of the booster vaccination intention to resist the mutated new virus and achieve herd immunity. In order to fill the above research gaps, we applied expectation confirmation theory to the study of COVID-19 vaccine booster shots to explore the factors influencing people’s vaccination intention. According to the existing literature and scale, we innovatively introduced the variable of perceived vaccination convenience to enhance the explanatory power of vaccination service convenience.

## 3. Research Model and Hypotheses

### 3.1. Research Hypotheses

#### 3.1.1. Perceived Performance

In this study, the perceived performance of vaccines included three aspects: perceived vaccine efficacy, perceived vaccination convenience, and perceived vaccine safety. Perceived vaccine efficacy and perceived vaccine safety are the product characteristics of the vaccine itself, while perceived vaccination convenience belongs to vaccine service. These three variables measure not only the cognition formed based on media information and other people’s evaluation, but also, more importantly, the psychological perception of people after COVID-19 vaccination.

(1) Perceived Vaccine Efficacy

Perceived vaccine efficacy refers to people’s perception of the efficacy or protection of COVID-19 vaccines. Some academics have found that vaccination is regarded as an unpleasant and risky experience for those who believe they are too weak to ward off viruses and diseases. On the contrary, people with high perceived vaccine safety would think vaccination is a more pleasant and less risky behavior [16]. According to expectation confirmation theory, the higher the perceived vaccine efficacy, the higher the expectation confirmation for COVID-19 vaccines. Therefore, we propose the following hypothesis:

**Hypothesis 1** **(H1):**  *Perceived vaccine efficacy has a positive effect on people’s expectation confirmation toward receiving the COVID-19 vaccine.*

(2) Perceived Vaccination Convenience

Perceived vaccination convenience is defined as the convenience degree of service people perceive during their COVID-19 vaccination experience. Previous research has shown that perceived convenience positively affects people’s attitudes toward this behavior [37]. When people find that they can access health care with less effort and lower cost, that is, more convenience, they will have a positive attitude toward it [38]. Before the experimental design, we conducted a small presurvey in which we interviewed respondents from different places. We found that some respondents living in both urban and rural areas of China delayed or refused booster shots because of the inconvenience of vaccination, but their reasons were quite different. Interviewees who lived in cities considered this inconvenience to be mainly due to working overtime frequently; the working hours being the same as the service hours of the vaccination site; hence, when they were off duty, the vaccination service was over; or the queue time at the vaccination site was too long, and they were reluctant to wait. For those living in rural areas, the geographical distance and round-trip expenses were the main reasons for the inconvenience. Therefore, we propose the following hypothesis:

**Hypothesis 2** **(H2):**  *Perceived vaccination convenience has a positive effect on people’s expectation confirmation toward receiving the COVID-19 vaccine.*

(3) Perceived Vaccine Safety

Perceived vaccine safety is defined as people’s perception of the safety and reliability of COVID-19 vaccines. The higher the perceived safety, the less people worry about the side effects of COVID-19 vaccines. Although serious adverse side effects of vaccination are rare, concerns and doubts about vaccine safety remain a major reason for vaccine hesitancy. Those who believe that vaccines have dangerous side effects may believe that vaccination is risky [17], resulting in a lower expectation confirmation level. Therefore, we propose the following hypothesis:

**Hypothesis 3** **(H3):**  *Perceived vaccine safety has a positive effect on people’s expectation confirmation toward receiving the COVID-19 vaccine.*

#### 3.1.2. Expectation Confirmation and Vaccination Intention for COVID-19 Vaccine Boosters

Expectation confirmation refers to an individual’s perception of the outcome as being in line with the original expectation [32]. In our model, vaccination intention is defined as people’s willingness to obtain a COVID-19 vaccine booster shot. In terms of vaccination time and immunization procedure, booster shots are additional vaccinations after full vaccinations. The vaccination intention for COVID-19 vaccine boosters can also be regarded as revaccination intention, which is equivalent to consumers’ “repurchase intention” in the commercial products field. Before people decide whether to obtain a COVID-19 booster shot, they have received at least one dose of the COVID-19 vaccine. The expected confirmation level of the COVID-19 vaccine, formed after people’s cognition of the vaccine, is influenced by their firsthand experience. According to expectation confirmation theory, when people’s expectations and needs for COVID-19 vaccine are met, their satisfaction will greatly increase. They will be more likely to complete additional vaccination on the advice of medical experts. Many studies in different fields have shown that expectation confirmation positively affects continuous intention.Therefore, we propose the following hypothesis:

**Hypothesis 4** **(H4):**  *People’s expectation confirmation toward receiving the COVID-19 vaccine has a positive effect on the vaccination intention for COVID-19 vaccine boosters.*

#### 3.1.3. Moderator Variables

(1) Health Consciousness

In this paper, we included health consciousness as a moderator variable. Health consciousness is defined as the degree to which individuals pay attention to health issues in their daily lives and the resulting motivation to improve their health [39]. The theory of collective action states that rational individuals give priority to their interests rather than collective interests in a large collective [40]. Since the COVID-19 pandemic, countries worldwide have been carrying out large-scale vaccination programs to achieve herd immunity as soon as possible. According to the theory of collective action, the collective good is nonexclusive and noncompetitive. Once herd immunity is achieved, it will benefit everyone regardless of whether the member has contributed to the herd immunity. This results in some people lacking the motivation to vaccinate against COVID-19 in order to achieve herd immunity. When people with higher levels of health consciousness are concerned about their health problems, they may be more sensitive to perceived vaccine safety and view vaccination as a risky choice. Therefore, we propose the following hypothesis:

**Hypothesis 5** **(H5):**  *Health consciousness negatively moderates the relationship between perceived vaccine safety and expectation confirmation.*

(2) Time Interval

We also included the time interval as a moderator variable. The time interval is a manifest variable, defined as the time interval between the last injection (nonbooster) and the present for fully vaccinated people. For example, if someone received two doses of the inactivated vaccine, with the second dose in July 2021, the time interval between March 2022 (when data were collected for this study) would be eight months. Medical experts have said that over time, after receiving the COVID-19 vaccine, some people’s antibody levels decline, and the protective effect of the vaccine is weakened. Experts recommended receiving a booster shot six months after full vaccination to maintain the protective effect. The situational crisis communication theory suggests that people have a higher level of trust in the government and other authorities after a major crisis or event. Previous studies have found that the public can benefit from effective publicity and communication during an influenza pandemic, which can help guide them to comply with recommendations during a public health crisis [41]. During this pandemic, we infer that the public will trust the advice given by vaccine experts. People’s belief that the vaccine efficacy may diminish over time will affect the level of the expected confirmation. Therefore, we propose the following hypothesis:

**Hypothesis 6** **(H6):**  *The time interval negatively moderates the relationship between the perceived vaccine efficacy and the expectation confirmation.*

### 3.2. Research Model

**Figure 1:**
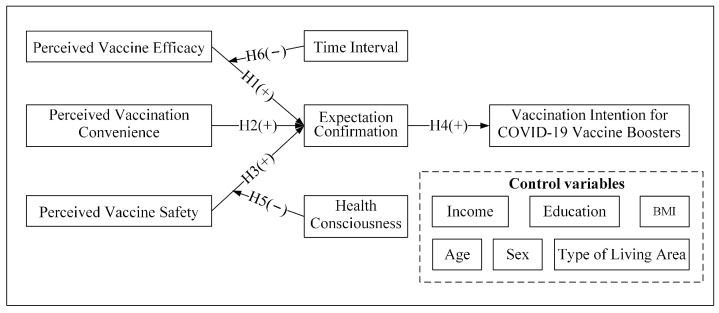
A research model of the factors of the vaccination intention for COVID-19 vaccine boosters.

As shown in Figure 1, based on expectation confirmation theory, this study used perceived vaccine efficacy, perceived vaccination convenience and perceived vaccine safety to measure people’s perceived performance of the COVID-19 vaccine. Furthermore, we introduced expectation confirmation theory and constructed a model of the factors of the vaccination intention for COVID-19 vaccine boosters. Control variables for the model included respondents’ sex, age, body mass index (BMI), income, education and type of living area. Previous studies have shown that the effectiveness of COVID-19 vaccine in normal-weight and obese groups is different, which may also lead to vaccine hesitancy in obese individuals [26], so we included the body mass index (BMI) as a control variable. Studies have shown that people’s perceived risk is related to whether they are in a risk area [42], which may potentially affect people’s expectation confirmation of the COVID-19 vaccine, thus reducing their vaccination intention. To explore whether living in the place where the COVID-19 cases occurred had a potential impact on their views on vaccines, we collected information on each respondent’s living area, which was divided into risk and nonrisk areas according to official announcements. We added the type of living area to the model as a control variable.

## 4. Materials and Methods

### 4.1. Variables and Measurement

The questionnaire consisted of the screening section, respondents’ basic information, and the main section. Qualified respondents were screened through the screening section. The basic information included demographic variables: sex, age, education level, body mass index (BMI), income, education, and type of living area.

As shown in Table 1, six latent variables and a manifest variable were measured in the main part, including twenty items. Latent variables included perceived vaccine efficacy (PVE), perceived vaccine safety (PVS), perceived vaccination convenience (PVC), expectation confirmation (EC), vaccination intention for COVID-19 vaccine boosters (VI), and health consciousness (HC). All the measures of the latent variables were adapted from the previous literature. The perceived vaccination convenience was adapted from an internet hospital study. Combined with the booster vaccination scenario, we adapted it for the items of this study. The manifest variable measured the time interval between the last injection (nonbooster) and the present for fully vaccinated people. The latent variables in the questionnaire all used the 5-level Likert scale, from strongly disagree to strongly agree. In the measurement items of our questionnaire, perceived vaccine safety was expressed negatively, which was processed before the subsequent model analysis to ensure consistency between variables. Although respondents had access to their vaccination records, we could not ensure that every respondent had this accurate vaccination information when filling in the questionnaire. On the other hand, people’s memories of previous vaccinations were likely to be vague. Therefore, we set the time interval as a categorical variable, and the options were: “less than 6 months”, “6–8 months”, “8–10 months”, “10–12 months”, and “more than one year”.

Expectation confirmation mainly includes three measurement methods: objective, inferred, and perceived. Perceived expectation confirmation measures consumers’ subjective evaluation of the difference after the consumption behavior between the expectation and the perceived performance [32]. The perceived type is the most commonly used measurement method because the perceived expectation confirmation considers the joint influence of prior expectation and perceived performance. In this study, we also adopted the perceived expectation confirmation.

### 4.2. Sample Size and Data Collection

#### 4.2.1. Sample Size

This study used questionnaires to investigate people’s vaccination intention for COVID-19 vaccine boosters and related factors. Before issuing the questionnaire, we evaluated the sample size required for the study. Full vaccination of COVID-19 vaccine referred to vaccination with one dose of the adenovirus vector vaccine, two doses of the inactivated vaccine, or three doses of the recombinant protein vaccine, excluding booster shots. Our study targeted people who had been fully vaccinated but had not yet received a booster shot. As of March 2022, 1239.57 million people had completed the full course of vaccination, among which 644.68 million people had completed the booster immunization [44]. The target population size is about 594.82 million people. According to Cochran’s formula [45], it can be calculated that at 5% margin level and 95% confidence level, the minimum sample size is 384.

#### 4.2.2. Data Collection

To ensure the quality and rationality of the questionnaires, we conducted a pilot survey before the formal collection. In this study, 30 respondents were recruited to fill in the questionnaires. According to their feedback, the items were easy to understand, and there was no large-scale missing value in the results. Therefore, questionnaires can be used to conduct formal investigations.

We distributed the questionnaires in two batches through various social media platforms in China. We collected 401 questionnaires from 18 to 28 March 2022, and 239 questionnaires from 22 to 25 October 2022. Participants were fully vaccinated adults. In this study, we eliminated the questionnaires of people who were not fully vaccinated according to the screening items. In addition, we excluded questionnaires with too short an answer time, inconsistent answers, and the incorrect selection of common knowledge. Finally, 572 valid questionnaires were collected, with an effective rate of 89.38%.

Table 2 shows the demographic information of the 572 respondents. This study collected data on the respondents’ sex, age, education level, the living area, and income level. At the same time, height and weight data were collected to calculate each person’s BMI. Among the participants, females accounted for 53.50%. Respondents aged 26–40 years were the largest group at 36.71%, with 36.54% and 23.95% aged 18–25 years and 41–60 years, respectively. Only 2.45% of respondents were aged 60 years or older. One reason is that there are fewer elderly people who can access the Internet and thus complete the questionnaire. The other reason is that the elderly suffer more from chronic diseases. Compared with the younger group, there were few older people who were fully vaccinated at this stage. Several empirical studies have published evidence that vaccine coverage is relatively low in older age groups [46,47]. More than 75% of the respondents had a bachelor’s degree or above. Respondents with a healthy weight (BMI between 18.5 and 23.9) accounted for 65.91%. In addition, among all respondents, 26.75% lived in risk areas when they filled out the questionnaire, while the other 73.25% came from nonrisk areas. The reason for this gap is that the criteria for the delineation of risk areas are very strict and cautious. Therefore, the population covered by risk areas must be much smaller than that of nonrisk areas.

### 4.3. Data Analysis

Partial least squares structural equation modeling (PLS-SEM) was used to analyze the research model. Our study used SmartPLS3.0 software for data analysis. SmartPLS3.0 is powerful software with an intuitive graphical user interface [48]. First, we performed a reliability and validity test on the questionnaire to assess the consistency and validity of the model. Then, we conducted the structural model tests.

A reliability and validity test is necessary to verify the quality of questionnaires. Reliability analysis measures the stability of the questionnaire test results, that is, the consistency of the results obtained, when the same object is repeatedly measured using the same method.

In the process of the structural model, the bootstrapping sampling method was adopted to test the hypotheses of the model; this method is very effective in studies with small data sets [49]. The bootstrapping sampling method can generate multiple training sets from the initial sample set. When a sample is selected, it is equally likely to be selected again and added to the training set. In the experiment, we set the sampling times to 5000. We conducted the main effect test and the moderating effect test.

## 5. Results

### 5.1. Reliability and Validity Test

Cronbach’s α coefficient and composite reliability (CR) are common indexes in the reliability test. In general, questionnaires with a Cronbach’s α coefficient and CR value above 0.7 meet the reliability requirements [50]. As shown in Table 3, the Cronbach’s α coefficients of each variable were all over 0.7, and the CR values were all over 0.8, indicating that our model has good internal consistency.

Validity refers to the degree to which a measurement tool can accurately measure the target subject. Structural validity includes convergent validity and discriminant validity. As shown in Table 3, the average variance extracted (AVE) in column 6 represents the convergent validity of the model. When the AVE is greater than 0.5, the model is considered to have good convergent validity [51]. The AVE values of all variables in the table were greater than 0.5, indicating that the convergent validity of our model met the requirements. Discriminant validity reflects the degree of difference among the latent variables [50,51]. As shown in Table 4, the diagonal elements of the discriminant validity matrix were the square roots of the AVEs. The square roots of the AVEs were greater than the correlation coefficients between this variable and other variables, indicating that our model had good discriminant validity.

### 5.2. Structural Model

#### 5.2.1. Main Effect Test

The variables involved in the main effect test were perceived vaccine efficacy, perceived vaccination convenience, perceived vaccine safety, expectation confirmation, and vaccination intention for COVID-19 vaccine boosters. The results of the main effect test are shown in Table 5. The t-statistics of hypotheses H1, H2, H3, and H4 were all higher than 1.96, and the p-values were less than 0.001. In terms of the characteristics of the vaccine itself, perceived vaccine efficacy (β = 0.266, *p* < 0.001) and perceived vaccine safety (β = 0.148, *p* < 0.001) had significant positive effects on expectation confirmation, and hypotheses H1 and H3 were supported. Regarding vaccination services, perceived vaccination convenience (β = 0.238, *p* < 0.01) also had a significant positive effect on expectation confirmation; so, hypothesis H2 was supported. Expectation confirmation (β = 0.586, *p* < 0.001) had a significant positive effect on the vaccination intention for COVID-19 vaccine boosters, and hypothesis H4 was supported. In addition, the type of living area (β = −0.013, *p* > 0.1) did not significantly affect people’s expectation confirmation.

#### 5.2.2. Moderating Effect Test

We added health consciousness and time interval variables to the original model to test their moderating effects. We examined the effect of the interaction term between health consciousness and the perceived vaccine safety and the interaction term between the time interval and the perceived vaccine efficacy on expectation confirmation. The results of the moderating effect test are shown in Table 6. Health consciousness (β = −0.133, *p* < 0.01) negatively moderated the positive effect of the perceived vaccine safety on the expectation confirmation; in addition, the time interval (β = −0.103, *p* < 0.05) negatively moderated the positive effect of the perceived vaccine efficacy on the expectation confirmation.

**Figure 2:**
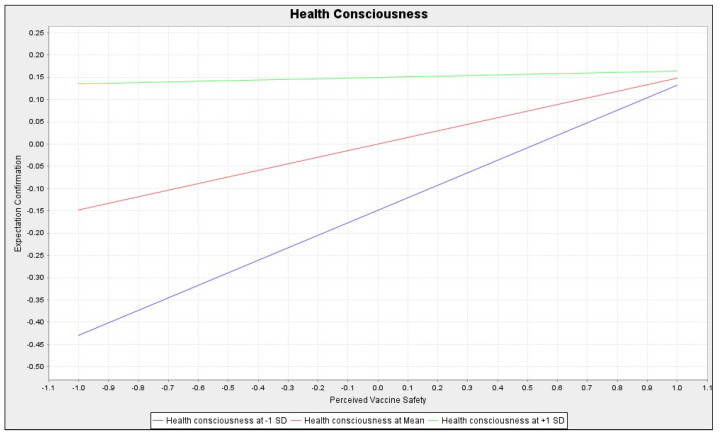
The moderating effects of health consciousness: Simple slope analysis chart.

**Figure 3:**
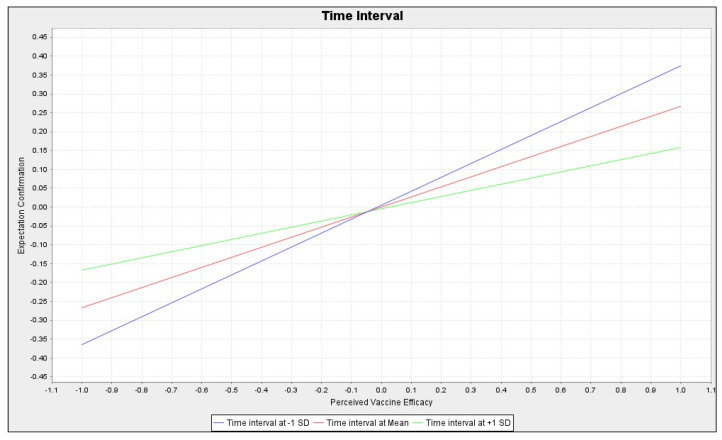
The moderating effects of the time interval: Simple slope analysis chart.

Figure 2 and Figure 3 visually illustrate these two moderating effects. Although perceived vaccine safety still had a significant positive effect on the expectation confirmation, this positive effect was weakened for people with high health consciousness compared to those with low health consciousness. As shown in Figure 2 (blue line -> red line -> green line), the slope became smaller. Therefore, hypothesis H5 was supported. Likewise, the positive effect of the perceived vaccine efficacy on expectation confirmation diminished slightly as the time interval from the last COVID-19 vaccine shot became longer. As shown in Figure 3 (blue line -> red line -> green line), the slope became smaller. Therefore, hypothesis H6 was supported.

## 6. Discussion and Contributions

### 6.1. Key Findings

First, expectation confirmation theory had a specific explanatory power for the factors of the COVID-19 vaccine booster vaccination intention. Our research model was based on expectation confirmation theory, and the relationship between variables was statistically significant. The model demonstrated that there was an expectation confirmation process for previous COVID-19 vaccines before people considered whether to obtain a booster shot. Our study is different from the previous studies on expectation confirmation theory for vaccination intention. Previous scholars explored the public’s process from motivation to intention, from intention to vaccination behavior, and finally to continuous vaccination [14]. It can be seen that the perspective of the previous study runs through the whole immunization program. However, in the reality that full vaccination rates are already high but booster vaccination rates are low, our study limits continuous vaccination to booster vaccination, which may have more realistic significance. The perspective of this study is only concerned with the process of how fully vaccinated people make the decision to vaccinate the booster shots.

Second, higher perceived vaccination convenience promoted the public’s intention to receive the COVID-19 booster shot. The previous literature used objective indicators such as distance or location to characterize the convenience of vaccination. In this study, the convenience of the vaccination service was measured in a perceptive way, which could better capture people’s subjective feelings and realize the transformation from perceived performance to expectation confirmation.

Third, our results showed that perceived vaccine efficacy and safety significantly affected people’s expectation confirmation level. As a medical product for the benefit of humankind, vaccine efficacy and safety are still crucial concerns. A previous study have considered the entire vaccination process holistically and used perceived usefulness to measure people’s overall attitudes toward vaccines [14]. In this paper, people’s evaluation of previous vaccination experience is divided into three aspects: perceived vaccine efficacy, perceived vaccination convenience, and perceived vaccine safety, so as to clearly understand the formation process of the public’s willingness to booster shots.

Fourth, we found the negative moderating effect of health consciousness on the relationship between perceived vaccine safety and expectation confirmation. Moreover, we found a negative moderating effect of the time interval on the relationship between perceived vaccine efficacy and expectation confirmation. Since people with high levels of health consciousness are very concerned about their health problems, they are more likely to view vaccination as a risky option. This may lead to a lower vaccination intention for booster shots. Regarding the moderating effect of the time interval, this shows that the views of medical experts will be listened to by the public. When people realize that antibody levels will decrease over time, such information could dampen confidence in the vaccine’s efficacy, thereby altering the level of the expectation confirmation.

### 6.2. Theoretical Contributions

This study introduced expectation confirmation theory to explain the formation process of the intention to receive COVID-19 booster shots. Expectation confirmation theory is widely used in consumer behavior research and continuous use of information systems but is rarely involved in vaccination. On the other hand, most vaccine-related research is based on the theory of planned behavior, conspiracy beliefs, and protection motivation theory [11,12,13]. This study introduces expectation confirmation theory into the vaccination intention research of booster shots, expanding the theoretical perspective on vaccination intention research.

This study introduced the variable of perceived vaccination convenience into the model. Several studies have focused on the impact of convenience on vaccination intention, but these studies used objective indicators to measure the convenience of vaccination. Based on the previous literature and scale, we adapted the perceived vaccination convenience to measure convenience from five dimensions. Our research improved the explanatory power of the convenience of the vaccination service and enriched the variables of the factor model of vaccination intention.

### 6.3. Practical Implications

The findings can provide a reference for public health institutions and help them understand the formation process of people’s intention to receive the COVID-19 vaccine booster to improve the coverage of COVID-19 booster shots. This study has practical significance.

In previous research [16,17], the perception of vaccine efficacy and safety perception was based on other people’s comments and media information, as a vaccine “word of mouth”, but the respondents in this study have been vaccinated with the COVID-19 vaccine. Their perception of vaccine efficacy and safety is more based on their feelings and judgment. In either case, the efficacy and safety of vaccines are important factors influencing the public’s intention to vaccinate. Therefore, public health institutions should ensure the quality and efficacy of vaccine products while promoting vaccines.

This study revealed the importance of the convenience of the vaccination service, and people’s subjective feelings about convenience came from various evaluations. Public health institutions should pay attention to the accessibility of vaccination services, such as increasing the number of vaccination sites, optimizing vaccination routes, and reducing vaccine costs.

The study also found negative moderating effects of health consciousness and the time interval, which could provide suggestions for public health institutions to improve booster coverage. Specifically, public health institutions can utilize big data intelligence to communicate more signals about vaccine safety to health-conscious groups. Hospitals and vaccination sites can inform people as soon as they become eligible to receive booster shots, avoiding a situation where people’s vaccination intention decreases due to longer time intervals.

### 6.4. Limitations and Future Directions

This study adopted questionnaires to collect samples. Although the sample size in this paper meets the requirement of minimum sample size, China’s population size is very large, and the limited sample size may have some selectivity bias. In addition, the age distribution of the samples mainly concentrates on youth and middle-aged adults, while the survey samples of the elderly were few. In future studies, we can collect larger samples to improve the generalizability of the study and consider increasing the number of samples of the elderly population through an offline questionnaire survey.

Furthermore, only perceived vaccine efficacy, perceived vaccine safety, and perceived vaccination convenience were included in this study to measure the perceived performance. In future studies, we can add other perceived performance variables to improve the explanatory degree of the model.

On the other hand, the “three Cs” model of vaccine hesitancy recommended by the World Health Organization incorporates three aspects: confidence, complacency, and convenience [52]. The perceived vaccination convenience in this study corresponds to “convenience”, and the perceived vaccine safety and efficacy correspond to “confidence”. The complacency factors were not present in this study because they were not associated with the perceived performance of the vaccine. In future studies, we can consider adding the complacency factors to improve the explanatory power of the model.

## 7. Conclusions

The COVID-19 vaccine booster has been shown to be effective in halting the rapid spread of the Omicron variant. Although the full coverage rate of the COVID-19 vaccine in China has reached a high level, the coverage rate of booster vaccination is still relatively low. Timely understanding of booster vaccination intention and its factors is crucial for public health. In this paper, we introduced expectation confirmation theory as the basis to construct a model of the factors of vaccination intention for COVID-19 vaccine boosters. A questionnaire survey was conducted to explore people’s vaccination intention for the COVID-19 vaccine and its factors. The vaccination intention for the booster shots is considered to be the intention to vaccinate “again”, influenced by the degree of expected confirmation of the previous vaccination experience. The higher the degree of expectation confirmation, the stronger the vaccination intention for the booster shots. The results showed that expected confirmation was positively influenced by perceived vaccine efficacy, perceived vaccine safety, and perceived vaccination convenience. In addition, for fully vaccinated individuals, the positive effect of perceived vaccine efficacy on expected confirmation was attenuated over time. Similarly, health consciousness negatively moderates the positive relationship between perceived vaccine safety and expectation confirmation. The main innovations of this study are the introduction of the expectation confirmation theory into the field of booster shots, the replacement of the single dimension measurement of convenience by the multidimensional perceived vaccination convenience, and the discovery of the moderating effect of the time interval. These findings can provide strong theoretical support for public health agencies. The limitation of this study is that the influencing factors of the research model are not fully considered. In future studies, we can explore the influence of other factors on the vaccination intention for the COVID-19 booster vaccine.

## Figures and Tables

**Table 1:** Variables, items, and their sources.

Variables	Name	Items	Sources
Perceived Vaccine Efficacy	PVE1	I believe the COVID-19 vaccine is effective in preventing the COVID-19.	[16,17]
	PVE2	I believe if I get the COVID-19 vaccine, I will be less likely to get the COVID-19.	
	PVE3	I believe the COVID-19 vaccine works in preventing the COVID-19.	
Perceived Vaccine Safety	PVS1	I worry about the short term side effects of the COVID-19 vaccine.	[16,17]
	PVS2	I worry that the COVID-19 vaccine might negatively affect my body.	
	PVS3	I worry that the COVID-19 vaccine might have unknown long term side effects.	
Perceived Vaccination Convenience	PVC1	I think it is very convenient to get the COVID-19 vaccine.	[27,39]
	PVC2	I think I am able to get the COVID-19 vaccine in a hospital or community health center.	
	PVC3	I think the place providing vaccination service is close to my home/work.	
	PVC4	I think the cost of COVID-19 vaccination is low (or no cost).	
	PVC5	I think that getting the COVID-19 vaccine could be helpful to decrease losses such as the physical damage and the loss of normal income due to quarantine or infection.	
Expectation Confirmation	EC1	My experience with the Covid-19 vaccine has been better than I expected.	[32,34]
	EC2	The vaccination service was better than I expected.	
	EC3	Overall, getting vaccinated against COVID-19 can meet demand beyond my expectations.	
Vaccination Intention	VI1	I am willing to receive vaccination for the COVID-19 vaccine boosters.	[43]
	VI2	I plan to get vaccinated for the COVID-19 vaccine boosters when the vaccine becomes available.	
	VI3	I will get vaccinated for the COVID-19 vaccine boosters as soon as it becomes available.	
Health Consciousness	HC1	I am aware of and very concerned about my health problems.	[39]
	HC2	I will try to manage and improve my wellness.	
Time Interval	TI	The time interval between the last injection (non-booster) and the present for fully vaccinated people.	–

**Table 2:** Demographic information of the respondents.

Variables	Value	Frequency	Percentage
Sex	Male	266	46.50%
	Female	306	53.50%
Age	18–25	209	36.54%
	26–40	210	36.71%
	41–60	137	23.95%
	>60	16	2.80%
Education	Junior high school degree and below	14	2.45%
	High school degree or GED	46	8.04%
	Associate degree	80	13.99%
	Bachelor degree	334	58.39%
	Master degree and above	98	17.13%
Income (RMB per month)	<1000	52	9.09%
	1000–3000	101	17.66%
	3001–5000	95	16.61%
	5001–8000	136	23.77%
	8001–10,000	78	13.64%
	10,001–15,000	44	7.69%
	>15,000	29	5.07%
	sorry, I would rather not to say	37	6.47%
BMI ^1^	underweight (<18.5)	52	9.09%
	healthy weight (18.5–23.9)	377	65.91%
	overweight (24.0–27.9)	108	28.88%
	obese (≥28)	35	6.12%
Type of living area	Risk area	153	26.75%
	Nonrisk area	419	73.25%

^1^ BMI: body mass index.

**Table 3:** Results of the reliability and convergent validity.

Variables	Factors	Standard Loadings	Cronbach’s α	CR ^1^	AVE ^2^
HC	HC1	0.877	0.71	0.873	0.775
	HC2	0.884			
VI	VI1	0.886	0.873	0.922	0.797
	VI2	0.892			
	VI3	0.901			
PVC	PVC1	0.786	0.82	0.874	0.583
	PVC2	0.792			
	PVC3	0.689			
	PVC4	0.729			
	PVC5	0.813			
PVE	PVE1	0.901	0.816	0.891	0.733
	PVE2	0.771			
	PVE3	0.89			
PVS	PVS1	0.919	0.925	0.953	0.87
	PVS2	0.953			
	PVS3	0.926			
EC	EC1	0.883	0.834	0.901	0.751
	EC2	0.834			
	EC3	0.882			

^1^ CR: composite reliability. ^2^ AVE: average variance extracted.

**Table 4:** The discriminant validity results of the research model.

	HC	VI	PVC	PVE	PVS	EC
HC	**0.881**					
VI	0.357	**0.893**				
PVC	0.426	0.472	**0.763**			
PVE	0.399	0.455	0.713	**0.856**		
PVS	0.083	0.262	0.202	0.262	**0.933**	
EC	0.403	0.587	0.538	0.546	0.254	**0.867**

Note: The diagonal numbers in bold are the square roots of the AVEs. The lower triangle is the Pearson correlation coefficient.

**Table 5:** Main effect test results of the research model.

Hypothesis	Paths	β ^1^	T-Statistic	Results
H1	PVE(+)→EC	0.266 ***	4.855	Supported
H2	PVC(+)→EC	0.238 **	3.438	Supported
H3	PVS(+)→EC	0.148 ***	3.837	Supported
H4	EC(+)→VI	0.586 ***	12.974	Supported

^1^*β*: standardized path coefficients. Note: *** *p* < 0.001; ** *p* < 0.01.

**Table 6:** Moderating effect test results of the research model.

Hypothesis	Paths	β ^1^	T-Statistic	Results
H5	HC*PVS(-)→EC	−0.133 **	3.191	Supported
H6	TI*PVE(-)→EC	−0.103 *	2.293	Supported

^1^*β*: standardized path coefficients. Note: ** *p* < 0.01; * *p* < 0.05.

## Data Availability

Not applicable.

## Abbreviations

The following abbreviations are used in this manuscript:

TI	Time interval
PVE	Perceived vaccine efficacy
PVS	Perceived vaccine safety
PVC	Perceived vaccination convenience
EC	Expectation confirmation
VI	Vaccination intention for COVID-19 vaccine boosters
HC	Health consciousness

## References

[1] WHO Coronavirus (COVID-19) Dashboard. https://covid19.who.int/.

[2] Chenchula S., Karunakaran P., Sharma S., Chavan M. (2022). Current evidence on efficacy of COVID-19 booster dose vaccination against the Omicron variant: A systematic review. J. Med. Virol..

[3] Burckhardt R.M., Dennehy J.J., Poon L.L.M., Saif L.J., Enquist L.W. (2022). Are COVID-19 vaccine boosters needed? the science behind boosters. J. Virol..

[4] Shekhar R., Garg I., Pal S., Kottewar S., Sheikh A.B. (2021). COVID-19 Vaccine Booster: To Boost or Not to Boost. Infect. Dis. Rep..

[5] SARS-CoV-2 Variants of Concern and Variants under Investigation in England. https://assets.publishing.service.gov.uk/government/uploads/system/uploads/attachment_data/file/1045619/Technical-Briefing-31-Dec-2021-Omicron_severity_update.pdf.

[6] Peiris M., Cheng S., Mok C.K., Leung Y., Ng S., Chan K., Ko F., Yiu K., Lam B., Lau E. (2022). Neutralizing antibody titres to SARS-COV-2 omicron variant and wild-type virus in those with past infection or vaccinated or boosted with mrna BNT162B2 or inactivated Coronavac vaccines(preprint). Res. Sq..

[7] Nemet I., Kliker L., Lustig Y., Zuckerman N.S., Erster O., Cohen C., Kreiss Y., Alroy-Preis S., Regev-Yochay G., Mendelson E. (2022). Third BNT162B2 vaccination neutralization of SARS-COV-2 omicron infection. N. Engl. J. Med..

[8] Galmiche S., Nguyen L.B.L., Tartour E., de Lamballerie X., Wittkop L., Loubet P., Launay O. (2022). Immunological and clinical efficacy of COVID-19 vaccines in immunocompromised populations: A systematic review. Clin. Microbiol. Infect..

[9] Zhang K., Wu X.X., Shi Y., Gou X.Q., Huang J.Q. (2021). Immunogenicity of H5N1 influenza vaccines in elderly adults: A systematic review and meta-analysis. Hum. Vaccines Immunother..

[10] Tohme R.A., Awosika-Olumo D., Nielsen C., Khuwaja S., Scott J., Xing J., Drobeniuc J., Hu D.J., Turner C., Wafeeg T. (2011). Evaluation of hepatitis B vaccine immunogenicity among older adults during an outbreak response in assisted living facilities. Vaccine.

[11] Patwary M.M., Bardhan M., Disha A.S., Hasan M., Haque M.Z., Sultana R., Hossain M.R., Browning M.H., Alam M.A., Sallam M. (2021). Determinants of COVID-19 vaccine acceptance among the adult population of Bangladesh using the health belief model and the theory of planned behavior model. Vaccines.

[12] Li L., Wang J., Nicholas S., Maitland E., Leng A., Liu R. (2021). The intention to receive the COVID-19 vaccine in China: Insights From Protection Motivation theory. Vaccines.

[13] Eberhardt J., Ling J. (2021). Predicting COVID-19 vaccination intention using protection motivation theory and conspiracy beliefs. Vaccine.

[14] Zhu W.L., Zou H., Song Y., Ren L.L., Xu Y.J. (2021). Understanding the continuous vaccination of the COVID-19 vaccine: An empirical study from China. Hum. Vaccines Immunother..

[15] Charron J., Gautier A., Jestin C. (2020). Influence of information sources on vaccine hesitancy and practices. Med. Mal. Infect..

[16] Nan X., Xie B., Madden K. (2012). Acceptability of the H1N1 vaccine among older adults: The interplay of message framing and perceived vaccine safety and efficacy. Health Commun..

[17] Nan X.L., Madden K., Richards A., Holt C., Wang M.Q., Tracy K. (2016). Message Framing, Perceived Susceptibility, and Intentions to Vaccinate Children Against HPV Among African American Parents. Health Commun..

[18] Karlsson L.C., Soveri A., Lewandowsky S., Karlsson L., Karlsson H., Nolvi S., Karukivi M., Lindfelt M., Antfolk J. (2021). Fearing the disease or the vaccine: The case of COVID-19. Personal. Individ. Differ..

[19] Tandy C.B., Jabson Tree J.M. (2021). Attitudes of East Tennessee residents towards general and pertussis vaccination: A qualitative study. BMC Public Health.

[20] Beraud G. (2021). Shortages Without Frontiers: Antimicrobial Drug and Vaccine Shortages Impact Far Beyond the Individual!. Front. Med..

[21] Bouchez M., Ward J.K., Bocquier A., Benamouzig D., Peretti-Watel P., Seror V., Verger P. (2021). Physicians’ decision processes about the HPV vaccine: A qualitative study. Vaccine.

[22] Latkin C.A., Dayton L., Yi G., Colon B., Kong X.R. (2021). Mask usage, social distancing, racial, and gender correlates of COVID-19 vaccine intentions among adults in the US. PLoS ONE.

[23] Yoda T., Katsuyama H. (2021). Willingness to Receive COVID-19 Vaccination in Japan. Vaccines.

[24] Gunes N. (2020). Parents’ Perspectives about Vaccine Hesitancies and Vaccine Rejection, in the West of Turkey. J. Pediatr. Nurs..

[25] Krasnicka J., Krajewska-Kulak E., Klimaszewska K., Cybulski M., Guzowski A., Kowalewska B., Jankowiak B., Rolka H., Doroszkiewicz H., Kulak W. (2018). Mandatory and recommended vaccinations in Poland in the views of parents. Hum. Vaccines Immunother..

[26] Townsend M.J., Kyle T.K., Stanford F.C. (2021). COVID-19 Vaccination and Obesity: Optimism and Challenges. Obesity.

[27] Vasudevan L., Baumgartner J.N., Moses S., Ngadaya E., Mfinanga S.G., Ostermann J. (2020). Parental concerns and uptake of childhood vaccines in rural Tanzania - a mixed methods study. Bmc Public Health.

[28] Yadete T., Batra K., Netski D.M., Antonio S., Patros M.J., Bester J.C. (2021). Assessing Acceptability of COVID-19 Vaccine Booster Dose among Adult Americans: A Cross-Sectional Study. Vaccines.

[29] Qin C., Wang R., Tao L., Liu M., Liu J. (2022). Acceptance of a third dose of COVID-19 vaccine and associated factors in China based on Health Belief Model: A national cross-sectional study. Vaccines.

[30] Hu T., Li L., Lin C., Yang Z., Chow C., Lu Z., You C. (2022). An analysis of the willingness to the COVID-19 vaccine booster shots among urban employees: Evidence from a megacity H in eastern China. Int. J. Environ. Res. Public Health.

[31] Neely S.R., Scacco J.M. (2022). Receptiveness of American adults to COVID-19 vaccine boosters: A survey analysis. PEC Innov..

[32] Oliver R.L. (1980). A cognitive model of the antecedents and consequences of satisfaction decisions. J. Mark. Res..

[33] Vijay T.S., Prashar S., Gupta S. (2018). Intention to provide online reviews: An expectation-confirmation model with review involvement. Pac. Asia J. Assoc. Inf. Syst..

[34] Bhattacherjee A. (2001). Understanding information systems continuance: An expectation-confirmation model. Mis Q..

[35] Ott J.J., Wiersma S.T. (2013). Single-dose administration of inactivated hepatitis A vaccination in the context of hepatitis A vaccine recommendations. Int. J. Infect. Dis..

[36] La Torre G., Mannocci A., Colamesta V., D’Egidio V., Sestili C., Spadea A. (2016). Influenza and pneumococcal vaccination in hematological malignancies: A systematic review of efficacy, effectiveness and safety. Mediterr. J. Hematol. Infect. Dis..

[37] Chi-Cheng C.-F.C., Yan C.-F., Tseng J.-S. (2012). Perceived convenience in an extended technology acceptance model mobile technology and English learning for college students. Australas. J. Educ. Technol..

[38] Valikodath N.G., Leveque T.K., Wang S.Y., Lee P.P., Newman-Casey P.A., Hansen S.O., Woodward M.A. (2017). Patient attitudes toward telemedicine for diabetic retinopathy. Telemed. e-Health.

[39] Li D., Hu Y., Pfaff H., Wang L., Deng L., Lu C., Xia S., Cheng S., Zhu X., Wu X. (2020). Determinants of patients’ intention to use the online inquiry services provided by internet hospitals: Empirical evidence from China. J. Med Internet Res..

[40] Olson M.J. (1965). The Logic of Collective Action: Public Goods and the Theory of Groups.

[41] Bickham S.B., Francis D.B. (2021). The Public’s Perceptions of Government Officials’ Communication in the Wake of the COVID-19 Pandemic. J. Creat. Commun..

[42] Chen Y.Y., Feng J.H., Chen A., Lee J.E., An L. (2021). Risk perception of COVID-19: A comparative analysis of China and South Korea. Int. J. Disaster Risk Reduct..

[43] Hong Y., Hashimoto M. (2021). I will get myself vaccinated for others: The interplay of message frame, reference point, and perceived risk on intention for COVID-19 vaccine. Health Commun..

[44] Official Website of China’s National Health Commission. http://www.nhc.gov.cn/.

[45] Cochran W.G. (1977). Sampling Techniques.

[46] Smith D.J., Hakim A.J., Leung G.M., Xu W., Schluter W.W., Novak R.T., Marston B., Hersh B.S. (2022). Covid-19 mortality and vaccine coverage — hong kong special administrative region, China, January 6, 2022–March 21, 2022. MMWR. Morb. Mortal. Wkly. Rep..

[47] Mistry S.K., Ali A.R.M.M., Yadav U.N., Huda M.N., Parray A.A., Mahumud R.A., Mitra D. (2022). COVID-19 vaccination coverage is extremely low among older population in Bangladesh: Findings from a cross-sectional study. Hum. Vaccines Immunother..

[48] Sarstedt M., Cheah J.H. (2019). Partial least squares structural equation modeling using SmartPLS: A software review. J. Mark. Anal..

[49] Gorondutse A.H., Hilman H. (1988). Does organizational culture matter in the relationship between trust and SMEs performance. Manag. Decis..

[50] Fornell C., Larcker D.F. (1981). Evaluating Structural Equation Models with Unobservable Variables and Measurement Error. J. Mark. Res..

[51] Anderson J.C., Gerbing D.W. (1988). Structural equation modeling in practice: A review and recommended two-step approach. Psychol. Bull..

[52] MacDonald N.E., SAGE Working Group on Vaccine Hesitancy (2015). Vaccine hesitancy: Definition, scope and determinants. Vaccine.

